# Understanding hospital antimicrobial prescribing decisions and determinants of uptake of new local antimicrobial prescribing guidelines in Laos

**DOI:** 10.12688/wellcomeopenres.20884.2

**Published:** 2024-09-12

**Authors:** Vilada Chansamouth, Anousone Douangnouvong, Peeyanout Thammavongsa, Xaysana Sombandith, Sommay Keomany, Sommana Rattana, Paul N Newton, Nicholas PJ Day, Paul Turner, Mayfong Mayxay, H. Rogier van Doorn, Elizabeth A Ashley

**Affiliations:** 1 Lao-Oxford-Mahosot Hospital-Wellcome Trust Research Unit (LOMWRU), Microbiology Laboratory, Mahosot Hospital, Vientiane, Lao People's Democratic Republic; 2 Centre for Tropical Medicine & Global Health, Nuffield Department of Medicine, University of Oxford, Oxford, UK; 3 Mahosot Hospital, Ministry of Health, Vientiane, Lao People's Democratic Republic; 4 Salavan Provincial Hospital, Ministry of Health, Salavan, Lao People's Democratic Republic; 5 Department of Healthcare and Rehabilitation, Ministry of Health, Vientiane, Lao People's Democratic Republic; 6 Mahidol Oxford Tropical Medicine Research Unit (MORU), Faculty of Tropical Medicine, Mahidol University, Bangkok, Thailand; 7 Cambodia Oxford Medical Research Unit (COMRU), Angkor Hospital for Children, Siem Reap, Cambodia; 8 Institute of Research and Education Development (IRED), University of Health Sciences, Ministry of Health, Vientiane, Lao People's Democratic Republic; 9 Oxford University Clinical Research Unit (OUCRU), Hanoi, Vietnam

**Keywords:** Antibiotic prescribing, antibiotic stewardship, hospital, guidelines uptake, qualitative study, Laos

## Abstract

**Background:**

Antimicrobial use in Laos is among the highest in Southeast Asia. The first Lao comprehensive antimicrobial prescribing guidelines have been available since 2021. This study explored the determinants of antibiotic prescribing decisions and how the new prescribing guidelines were being used.

**Methods:**

In August 2022, in-depth interviews were conducted with 16 Lao prescribers from two hospitals. Participants were questioned about their prescribing behaviours, attitudes to guidelines, how they learned about the guidelines and factors influencing their uptake. The interviews were audio-recorded, transcribed, and translated into English. Thematic analysis of the transcripts was conducted.

**Results:**

Lao prescribers considered multiple factors before deciding to prescribe antibiotics to their patients. The most common factor was based on the clinical judgement of the prescribers. Lack of certain antibiotics and turnaround times of laboratory results were the main challenges to prescribing antibiotics appropriately. The majority of participants were satisfied with the guidelines, regarding them as comprehensive, simple and convenient. However, most participants admitted that they did not access the guidelines very often. The main reason was that they could remember the treatment recommendations because they treat similar diseases on a daily basis. Improving antibiotic knowledge was the most common recommendation in order to improve the appropriate use of antibiotics. Raising awareness of the guidelines and promoting their use should also be considered. In addition, heads of the wards, and policy and implementation leaders, should support, monitor and feedback their use to encourage all prescribers to follow the guidelines.

**Conclusions:**

Several factors contribute to enhancing appropriate antibiotic prescription. Key factors for improving antibiotic prescription include enhancing prescribers' clinical knowledge, ensuring access to essential antibiotics, and updating guidelines regularly. Health leaders must get involved to promote their use.

## Plain language summary

In Laos, antibiotic use is high compared to other Southeast Asian countries. In 2021, the first guidelines for prescribing antibiotics were introduced in Laos. This study aims to explore what influences doctors’ decisions in prescribing antibiotics and how they used the new guidelines.

In August 2022, we conducted in-depth interviews with 16 doctors in two Lao hospitals. We asked them how they decided which antibiotics to give, what they thought about the guidelines, how they found the guidelines and what could make them use the guidelines more. We recorded, transcribed, and translated the conversations. Then, we identified common themes and patterns.

Before giving antibiotics, doctors in Laos considered many things. The most important thing was their own judgment based on their medical knowledge. Not having some antibiotics and waiting long time for the laboratory results were the main issues that made it challenging for doctors to prescribe antibiotics. Most interviewees liked the guidelines. They found the guidelines easy to understand and useful. Many of them said that they did not use the guidelines a lot. The main reason was that they remembered the treatment recommendations because they treat similar diseases every day. The most common suggestion to use antibiotics better was to learn and understand more about them. Also, leaders of hospital departments and those in charge of making rules should help, keep an eye on the use, and give feedback to make sure everyone who prescribes antibiotics uses the guidelines.

To make sure doctors prescribe antibiotics better, they need to know and understand more about infectious diseases, have easy access to essential antibiotics, and regularly update the guidelines with support from the leaders.

## Introduction

Antimicrobial use (AMU) is one of the key drivers of antimicrobial resistance (AMR) ^1^. The use of antimicrobials in the human healthcare sector has been shown to be influenced by several factors including previous clinical experience, physicians’ knowledge and attitudes ( *e.g.* ignorance, complacency), fear of harming patients, lack of autonomy ( *i.e.* following the decision of seniors), demands of patients and families, other patient-related factors, healthcare-related factors, the influence of pharmaceutical companies, and the cost of medications ^2– 4^. The appropriateness of AMU in hospitals could be improved with comprehensive and up-to-date antibiotic use guidelines. Maina *et al*. described that the prevalence of the appropriate use of antibiotics in settings with treatment guidelines was higher than where guidelines were absent (14% *vs.* 33%) ^5^. However, previous reports have emphasized that guidelines alone might not be enough to assure the appropriate use of antimicrobials. Combined approaches such as explicit and appropriate guideline implementation strategies, engagement with prescribers, antimicrobial stewardship programmes including restrictive policies, such as not allowing certain prescriptions to be dispensed without consultation with specialists or evidence of an infection requiring that agent; or prescribers not getting paid for their services if they did not follow the guideline recommendations ^6^ have been proposed to reduce the inappropriate use of antimicrobials ^5– 8^.

Laos is a lower middle-income country, bordered by Cambodia, China, Myanmar, Thailand and Vietnam. Laos began participation in global point prevalence surveys (PPS) of hospital antimicrobial use in 2017, with baseline data of hospital antimicrobial use as high as 71% between 2017 and 2020 among six general hospitals across the country ^9^. This percentage was higher than other low- and middle-income countries and high-income countries globally ^10, 11^. Laos has had treatment guidelines available for some specific diseases such as malaria, tuberculosis, and sexually transmitted infections for several years. The latest national standard treatment guidelines (STG) were released in 2012. These contained information on clinical characteristics of diseases, diagnoses and treatment recommendations. Infectious diseases were a part of the STG but the antibiotic treatment recommendations were only available for some diseases ^12^. A qualitative study to understand factors influencing uptake of the WHO pocketbook of hospital care for children in Laos showed that there were several key factors affecting uptake of the guidelines, including the completeness of the guideline, unclear Lao translation for some sections, the fact that guidelines were not always physically available, lack of training in their use, and lack of trust ^13^. The first comprehensive Lao antimicrobial prescribing guidelines for adults and children were introduced in January 2021 ^14, 15^. These guidelines were developed specifically to fill the gaps mentioned above. The guidelines were developed based on epidemiology of infectious diseases in Laos and local antimicrobial susceptibility, as well as international recommendations. They were made available for all prescribers in six general hospitals across Laos following a workshop on how to use them, as part of a stepped wedge cluster randomized controlled trial to evaluate the impact of guidelines delivered via a mobile phone application versus pocket book ( NCT04914793). The baseline data for antimicrobial prescription rate in six hospitals before introduction of the guidelines was 73% (95% CI, 70%-76.5%) among inpatients and 27% (95% CI, 25.8%-28.9%) among outpatients in the first survey round of 2021. Antimicrobial prescribing percentage increased to 79.6% (95% CI, 75.7%-83%; p=0.02), and 30% (95% CI, 28.4%-32.2%; p=0.01) among in- and outpatients, respectively, one year (the first survey round of 2022) after the introduction of the guidelines ( https://livedataoxford.shinyapps.io/amulaos/). One probable reason could be due to the peak of Covid-19 pandemic in Laos during 2021 and 2022. This increase of antibiotic use was globally described during Covid-19 pandemic ^16^. Therefore, this study aimed to explore the determinants of antibiotic prescribing decisions and how these new Lao antimicrobial prescribing guidelines were used.

## Methods

### Ethics and consent

Ethics approval was obtained from both the University of Health Sciences Ethics Committee, Ministry of Health, Laos (reference number: 318/REC, date 22 April 2022) and the Oxford Tropical Research Ethics Committee (OxTREC), University of Oxford, United Kingdom (reference number: 510–22, date: 02 April 2022). Written informed consent was obtained from all participants.

### Study design and settings

This qualitative study was designed to evaluate prescribers’ perceptions of the new guidelines and factors influencing their use at the sites participating in the stepped wedge cluster randomised trial outlined above. In-depth interviews were conducted in two general hospitals (one central hospital [450-beds] and one provincial hospital [70-beds]). These two hospitals were purposively selected as representative of a central referral hospital and a rural hospital in Laos. Prescribers in both hospitals had access to the new local antimicrobial prescribing guidelines in both pocketbook and mobile phone application versions and participated in the workshop of how to use these guidelines.

### Participants

Participants in this study were purposively selected based on seniority and their specialties (prescribing antibiotics, from infectious disease departments or intensive care units, pediatric *vs.* adult). We focused on prescribers who prescribed antibiotics at least three times a week on average and who had access to the local antimicrobial prescribing guidelines, either pocketbook or mobile phone application versions or both, and were willing to give written informed consent to participate. The possible target prescribers were listed based on the above-mentioned criteria before the invitations were distributed. The purposive selection allowed us to capture a range of different perspectives.

The eligible participants were invited to join the in-depth interviews, including senior doctors and juniors/residents in a 1:1 ratio. There is no formal definition of junior and senior physicians in Laos. In general, doctors who are less than 40 years old, are classified as early career, 40–50 years old as mid-career and >50 years old as late career. It is more complex when it comes to “senior *vs.* junior”. In this study, junior was defined as doctors/prescribers aged <40 years old or with medical experience of 10 years or less. Senior was defined as doctors/prescribers aged ≥40 years old and with medical experience of more than 10 years. 

### Study procedures and data collection

Invitation letters were sent to those who met the inclusion criteria. Written informed consent was obtained if the prescriber agreed to participate. Additional invitations were sent to new target interviewees in the list if the previous one declined the invitation. Two in-depth interviews a day were performed in August 2022, taking a maximum of one hour and a half per interviewee. Interviews were led by the first author in the Lao language and conducted based on the discussion guide (extended data 1). The in-depth interviews were performed face-to-face in a quiet and private room by the first author (VC) with the assistance of the second author (AD). Basic demographic information of the participants (age, sex, title (staff, residency), current specialty) was collected. The interview topics (see topic guide on extended data 1) focused on their experience of antimicrobial prescribing in their hospitals and their experience using the new local antimicrobial prescribing guidelines. Participants were questioned about their prescribing behaviours, attitudes to guidelines, how they learned about the guidelines and factors influencing guideline uptake ^17^. The interviews were audio-recorded, and later transcribed and translated into English by a professional translation company.

### Data analysis

First, second and third authors reviewed the transcripts for accuracy and completeness and discussed the content. Thematic analysis of the transcripts was conducted. Both inductive and deductive approaches were used to analyse the data. NVIVO software (version 14) was used to assist in coding and handling data. Codes were added, then discussed and modified among the authors. Themes were generated after agreement was reached for the codes. Main themes and sub-themes were identified based on the richest codes. Those themes with less supportive codes were combined as one theme.

## Results

The in-depth interviews were conducted in August 2022 with 16 interviewees (eight from each hospital). Among the participants, 12 (75%) were female, the median [interquartile range (IQR)] age was 36 (31–48) years and the median (IQR) years of medical experience was nine (6–18) years with half of them recognized as seniors. Four (25%) participants were from paediatric wards, four (25%) from adult general internal medical wards, four (25%) from intensive care units, three (19%) from infectious disease wards and one (1%) from a surgery ward (extended data 2). Nearly all participants had access to both paper-based and app-based Lao antimicrobial prescribing guidelines, with only one participant having access solely to the app-based guideline.

### Factors influencing antibiotic prescribing decisions

Prescribers shared their opinions about how they prescribed antibiotics in their facilities. They were asked what factors they considered before deciding to prescribe, including how they selected antibiotics, route of administration and why they might combine antibiotics (extended data 1). The majority of participants explained that they prescribed antibiotics based on the patient’s characteristics obtained from asking for the history of illness, underlying diseases, physical examination findings, and whether patients were in a severe condition. Suspected diseases and likely source of infection were also commonly mentioned as important factors for them to decide whether to start antibiotics, or which antibiotic or route of administration should be selected. Most of the time these factors influencing prescribing decisions took into account laboratory results, either complete blood count (CBC) or specimen culture result ( *e.g.*, blood culture). Most of the time, participants considered multiple factors before deciding to start antibiotic therapy. These were similar between participants in central and provincial hospitals. A few interviewees mentioned that sometimes they also prescribed antibiotics based on their habit or medical experience of treating patients consulting with the same disease ( *e.g.* pneumonia) when they already have a good idea what the causative pathogens are and what antibiotics need to be given. This was mentioned more commonly among participants from the provincial hospital than those from the central hospital. Nearly half of the participants said that they prescribed antibiotics based on treatment/antibiotic use guidelines or textbooks, mostly to decide doses and treatment duration. Four main themes emerged from analysis of this content to describe how prescribers prescribed, including prescribing based on clinical judgement, following what has been done before, following specialist recommendations, and prescribing due to a precautionary approach ( Table 1).

**Table 1.** Themes of factors influencing antibiotic prescribing from in-depth interviews with 16 prescribers in one central and one rural hospital in Laos.

Themes/sub-themes (number of participants/number of quotations)	Quotations (participant ID)
Prescribing based on their clinical judgement (15/57): 1. Patient characteristics (14/36) 2. Suspected disease/causative pathogens/ source of infection (12/17) 3. Antibiotic effects (2/2) 4. Previous antibiotic use by the patient (2/2)	“ *It will be based on two things: first, on the clinical [symptoms/physical examination] of the patient. After 3–5 days of medicines, we recheck if the patient is getting better or not; our initial diagnosis is correct or not. Second, we rely on the lab [laboratory] results. If the medicines we initially provided is not the right one, then we’ll need to change medicine based on the laboratory report*” (Participant 1, senior, medical ward, rural). *“Before we decide to prescribe antibiotics, we need to see if it is an infection. Most of the time, we search for the source of infection by information from the family. Most importantly is physical examination, because for myself and I believe for other doctors too, we need to know the source of infection before giving any antibiotic…. Above all, we cannot forget culture every single time, at least hemoculture each time”* (Participant 8, senior, intensive care unit, urban). “ *If patients present with a severe condition, with any body organ being affected badly or lightly, then the medicine will be based on the class of the antibiotics, or based on how severe the body organ is affected. These should be okay*.” (Participant 7, junior, medical ward, urban). “ *We also have to look into the choice of medicine. For example, for a case of pneumonia, we’ll have to see which bacteria is it. We’ll choose the lowest level [narrow spectrum] of medicine for the particular age group of the child, based on which bacteria is the common cause. We will not jump to a higher level [broad spectrum] of medicines*.” (Participant 3, junior, paediatric ward, urban). “ *Normally in my ward, we will base on the disease of the patient. For example, appendicitis, we would give ceftri [ceftriaxone] before surgery, cloxacillin for abscess, and ampicillin for normal injury*” (Participant 10, junior, surgical ward, rural). “ *Those with respiratory issues, like a patient with previous pneumonia, comes to hospital because of breathing difficulty, we examine and find lung crepitation, then we’ll immediately give antibiotics because this patient has previously received antibiotics. It’s chronic*” (Participant 12, junior, intensive care unit, rural).
Prescribing based on the existing resources (13/15): 1. Following laboratory results (11/13) 2. Following choice of available antibiotics in hospital (2/2)	“ *Patients who are admitted in our ward must have CBC tested urgently. If it shows high white blood cells, then undoubtedly, it’s an infection and would first need antibiotics*” (Participant 5, senior, medical ward, urban). “ *Before prescribing antibiotic for a patient, I mainly refer to the result of white blood cells of the patient. For instance, whether the level of white blood cells is high or too low in case the patient also has fever. A second criteria is the culture result, if it comes out positive. If not based on the above-mentioned reasons, then it would be mainly based on the CBC result and the condition of the patient*” (Participant 13, senior, medical ward, rural). “ *The fundamental of giving antibiotics is based on the availability of medicines in the hospital*” (Participant 16, junior, paediatric ward, urban).
Prescribing based on habit (7/9)	“ *We rely on the prescribing app, the patient’s condition, and our habits. For instance, when the patient has respiratory distress, for example, patient has pulmonary oedema, which there’s no need for antibiotic. Sometimes it’s a habit to start antibiotic thought the patient doesn't have fever, white cell counts are not high, but antibiotics are still given. Even if we don’t give the patient antibiotics, other doctors would give antibiotics out of habit.”* (Participant 9, junior, medical ward, rural). “ *For cases that we give many types of medicines [combined antibiotics], is for those who have gastric perforation, for example. Like this, we need to give many medicines…. because we follow what we have been practicing*” (Participant 10, junior, surgical ward, rural). “ *I use 2–3 types of antibiotics in case of infections in newborns, like ampi [ampicillin] and genta [gentamicin]. If still not confident, I would add cloxa [cloxacillin] for infection of newborns of late onset at home. When we’re not sure what the bacteria is so we give them 3 types of medicine*” (Participant 14, senior, paediatric ward, rural).
Prescribing due to precautionary approach (2/3)	“ *We will immediately give IV for patients admitted because we don’t want them to stay in the hospital long. If patients respond to our medicine, approximately three days then they want to leave hospital and we can continue with oral medicine....... First, we avoid all the people [relatives] who come and look after the patients. Two, the bacteria within the hospital, so we want to speed up [treatment process] so they can go home fast.*” (Participant 5, senior, medical ward, urban).
Following specialist recommendations (1/2)	“ *We would not give antibiotics for digestive system which are not infected, like GI [gastrointestinal] bleeding. After GI tract examination and discussion with other doctors [GI specialist], antibiotic is suggested, then we’ll prescribe antibiotics*” (Participant 4, senior, intensive care unit, urban).

### Challenges to appropriate antibiotic prescribing

Prescribers revealed that sometimes they have faced difficulties prescribing antibiotics appropriately. Lack of antibiotics in their settings was commonly mentioned among participants from central hospitals, including several important antibiotics that were not available in the country. Some said that the lack of antibiotics in their hospitals was not a big problem as patients’ relatives could always find the antibiotics required in private pharmacies.

“ *Currently, what is happening is that the culture results show MRSA [methicillin-resistant* Staphylococcus aureus *], and we want vanco [vancomycin] but it’s not available. We have to buy it elsewhere, like other hospitals which sometimes it is available but sometimes not*” (Participant 2, junior, paediatric ward, urban).

“ *We sometimes have to use lower effect medicines [narrow spectrum]. For example, ampho [amphotericin B] which is not available in Laos, is expensive and need to buy from Thailand. If ampho must be used but we don’t have it and the patient cannot afford it, then we have to change to an oral medicine*” (Participant 7, junior, medical ward, urban).

“ *If antibiotic is not available in the hospital, just go buy it from outside. It is easy to find*” (Participant 5, senior, medical ward, urban).

Perceived long turnaround time of laboratory results was another problem raised by some participants, mostly from provincial hospital. Some mentioned that they realized that the culture could take time, therefore, they usually did not wait for the laboratory results to decide whether they should have started antibiotics or not, or to choose which antibiotics they should have prescribed. Sometimes the microbiological results came too late for the prescribers to switch the antibiotic to a more appropriate one as the patient might have gone home or died. Here are examples of how some participants expressed their experience of perceived delayed microbiological results affecting how they prescribed antibiotics.

“ *We start with the highest antibiotic [broad spectrum] first until the report [culture result] comes then we reduce it. There was an ICU case that was given mero [meropenem] and vanco [vancomycin], until the report [culture result] came out showing staph [*Staphylococcus *] for which cloxa [cloxacillin] can be used. To that point, it was already over 10 days and only two days remaining [to complete the course of meropenem and vancomycin], so we continued with the same medicines without changing to cloxa [cloxacillin]*” (Participant 3, junior, paediatric ward, urban).

“ *Mostly the lab [laboratory] would provide us the rapid test results like CBC. We know that the hemoculture takes some time so we don’t wait for that*” (Participant 14, senior, paediatric ward, rural).

“ *Labs [laboratory] are not enough/adequate and not as timely as needed. Sometimes, we get the result after patients are released from the hospital or the patient has already passed away. Sometimes, the patients have already gone home, and we’re unable to give them medicine [in time]*” (Participant 15, junior, intensive care unit, rural).

Another challenge which was revealed by some junior prescribers was a difference of opinion on prescribing decisions from and between their senior(s). This was raised in both central and provincial hospitals equally. However, most of them mentioned that informing and discussing the decisions with seniors was essential because they had to make sure that they would have their full support if something went wrong, and their seniors could give different views as they had more experience.

“ *During working days/hours, many staff decide which medicine to give to the patient, but there’s a challenge because of different views. For instance, one professor [senior] wants to give a particular medicine while another professor [senior] wants to give a different one. Eventually, we would choose the decision of the most senior doctor. If the most senior doctor is not present, then the next senior one would decide. Hence, doctors who newly graduated wouldn’t have a say in the decision making*” (Participant 16, junior, paediatric ward, urban).

“ *Most of the time if they [seniors] prescribe, then I’ll follow the decision. But there are times when I question like: what if an infant aged 1–2 years presents with pneumonia with common bacteria, then the senior prescribes azithro [azithromycin] but instead I want to give amox [amoxicillin]. In this situation I would question to see if they have any reason for that or it is based on their experience, this medicine [azithromycin] is more effective. So, I don’t understand what they think. Sometimes I question, sometimes I don’t. But if you ask how I manage my thoughts... well, I let it go if I don’t want to have any problems*” (Participant 2, junior, paediatric ward, urban).

A few participants described their challenges in managing patients on inappropriate previous antibiotics where they had to follow the initial prescribing decision, or with specific patient characteristics such as allergic reaction to some antibiotics or having chronic disease, or who could not afford the cost of antibiotic. When asked where they search for help if they had a question relating to antibiotic therapy, most of participants said that they checked the information in textbooks/research articles/internet as well as asking opinions from seniors/colleagues. Some preferred to only ask seniors/colleagues as this was quicker.

“ *If the ward [ward doctor] has been giving the medicine for 1–2 days, then we’ll have to continue using it. This is another reason that we cannot avoid. If they’ve been given meropenem, then we can’t go back to the old medicine as we are not sure if patients would get better or get worse*” (Participant 8, senior, intensive care unit, urban).

“ *We have to discuss with the family first before change and adjust the medicine: we currently don’t have this medicine in the hospital and need to purchase from other hospital or outside pharmacy. If the patient’s family say they cannot afford to buy the medicine, we’ll need to find a way out*” (Participant 4, senior, intensive care unit, urban).

“ *I mainly discuss with my colleagues for what we’re not sure. We cannot decide alone. we have to jointly decide for a particularly difficult case to be accountable. We have to ensure safety first*” (Participant 7, junior, medical ward, urban).

### Uptake of the new Lao antimicrobial prescribing guidelines

The first comprehensive antimicrobial prescribing guidelines in Laos were distributed to all prescribers in these two hospitals in 2021. These in-depth interviews focused on prescribers’ perceptions of these new guidelines. Only a few themes emerged when asking when the participants used antibiotic prescribing guidelines. Nearly all participants said that they used the guidelines when they were not sure about the dose and duration of the antibiotic therapy, especially when patients had kidney problems. Some participants checked the guidelines when they were facing uncommon situations. Choices of antibiotics were also mentioned; for example, the participants looked for alternative antibiotics because what they wanted to prescribe was not available. The main reason for not consulting the guidelines was already remembering them by heart, a trend observed in both provincial and central hospitals. A participant also said that she/he did not use guidelines often because s/he followed what s/he has been doing in the ward or the recommendation from senior colleagues, another participant said that s/he was not quite sure how to use the guidelines (mobile application) ( Table 2).

**Table 2.** Understanding how Lao doctors used the first comprehensive antimicrobial prescribing guidelines.

Themes (number of participants/number of quotations)	Quotations (participant ID)
**When did you access Lao antimicrobial prescribing guidelines?**	
Searching for dose and duration of antibiotic treatment (15/16)	“ *I occasionally open the app because I always see patients and use the medicines often. I’ll open it for special cases, like patients with kidney failure where we would need to adjust medicine dose. Therefore, we have to check on the appropriate dose for the level of kidney issue so that it doesn’t affect the kidney*” (Participant 6, senior, medical ward, urban). “ *Most of the time, it’s when the patient has kidney issues and I’d like to provide the correct dose for kidney. Doses in general, I don’t really refer to it [guidelines], because I use [medicines] often and I can remember. But I need to check the dose for patients with kidney and liver disease*” (Participant 2, junior, paediatric ward, urban). “ *I look at what antibiotics to give, alternative medicine but I mostly check doses and duration of treatment*” (Participant 15, junior, intensive care unit, rural).
Facing uncommon situation (5/5)	“ *I mostly use the guidelines when I encounter a problem in treating a patient. For instance, we find low white blood cell on day 3, 4 or 5 of dengue fever; but on day 6, the level of white blood cells turns so high. Sometimes we are not sure about this strange situation whether patient has sepsis or not*” (Participant 13, senior, medical ward, rural). “ *I already remember most local infections… Let’s say, I use it [guidelines] when I encounter issues*” (Participant 11, senior, paediatric ward, rural).
Searching for choice of antibiotic therapy (4/4)	“ *Alternative medicine. For instance, if the hospital runs out of a medicine, then an alternative medicine can be given. The district hospital would call to consult us about the medicine based on the condition of the patient. If the medicine is not available, we’ll recommend them to give an alternative medicine based on the app*” (Participant 9, junior, medical ward, rural).
**Why did not you access Lao prescribing guidelines often?**	
Facing the same diseases and already remember the recommendations (9/9)	“ *I mostly do not open the book [guidelines] for the disease which I remember, for example sepsis, I already remember. As we know for baby aged less than 2–3 weeks, we use ampi [ampicillin], genta [gentamicin]. The dose is 50–100, let say we decided to use 50 …*” (Participant 14, senior, paediatric ward, rural). *“As I said, I opened the pocketbook once or twice. It says to use ceftri-azithro [ceftriaxone – azithromycin] and I then remember it. When there’s a case of pneumonia again, I will use the same medicines in the same dose, like this over and over again”* (Participant 9, junior, medical ward, rural).
Prescribing based on habit (1/1)	“ *Well, in our ward, we just follow what we have been doing in terms of giving medicines*” (Participant 10, junior, surgical ward, rural).
Not sure how to use it (1/1)	“ *I have to admit that I have never opened the pocketbook version of the guidelines, I have occasionally used the mobile phone application version …. But I do not understand if I should type the name of the antibiotic agent and then information comes up, or I have to go to each organ system [options to be selected in the application] and then I could find the information of the medicine*” (Participant 3, junior, paediatric ward, urban).

When asking participants to share their opinions on how their colleagues used the guidelines, the majority of them mentioned that they have seen their colleagues access the guidelines but not often. Some participants mentioned that personality was important as some colleagues just did not like reading or just followed what they have been doing in the past. Another reason for using guidelines less frequently could be because they have already remembered the guidelines or how to prescribe. Workload was also mentioned. Few participants mentioned about other guidelines/textbooks which were more commonly used in their areas.

“ *We have discussed often in our ward, especially our ICU ward. We’ve discussed when antibiotics should be used or when we have faced difficult/complex disease like rabies or tetanus. We check the guidelines together to find out which antibiotics we should use because the guidelines have everything*” (Participant 13, senior, medical ward, rural).

“ *It is a habit. For example, if patient comes with pneumonia or meningitis, doctors will prescribe the same medicines that they’ve used often which make the patient get better. Unless the patient doesn’t get better, then they will check the guidelines/textbook to see whether the medicines they prescribe are correct or not. It’s a habit. I’ve also done this before*” (Participant 3, junior, paediatric ward, urban).

“ *They don’t open it [guidelines] because their work is overloaded. For instance, in other countries, like Thailand, their doctors are on duty for eight hours and they take a rest. For us, we work from 8 o’clock of today to 8 o’clock of the following day. Some people are on duty three days in a row without rest. When they have little time, they want to rest their eyesight*” (Participant 11, senior, paediatric ward, rural).

“ *We use Harrison’s [textbook] in our general internal medicine wards. When we discuss with our seniors/professors, we have to refer to that book. Each senior/professor prefers a different textbook. Me, as senior, I encourage people to use this guideline [antibiotic prescribing guidelines]*” (Participant 1, senior, medical ward, rural).

The participants were also asked to share their opinions on the guidelines. Most participants mentioned that the guidelines were appropriate. The majority of participants said that they preferred the mobile phone application version to the pocketbook version as it was convenient and easy to search for the information. A few claimed that the pocketbook version suited them better as they did not feel comfortable with technology. When asking about their ideal guidelines or what they wanted to improve in these guidelines, eight out of 12 participants shared their views that they already agreed with these current guidelines. Some proposed different searching options ( *e.g.*, search by antibiotic agent or by pathogen) and updating guidelines every one to three years was suggested.

### Factors influencing better antibiotic prescribing and guidelines uptake

Participants proposed that improving and updating knowledge on antibiotic therapy were important to support appropriate use. This should not only be for prescribers in hospitals but also medical students. Engaging and raising awareness on good use of antibiotics could also support prescribers to prescribe more appropriately. Some participants suggested that restricting and monitoring the use of antibiotics could be effective solutions.

“ *Overseas, they have control measures on the use of antibiotics. If they were to order carbapenem for example, general practitioners could order use for 2–3 days, if more than that they would need to consult the infectious disease doctor. The pharmacy will not dispatch the medicine if they cannot define these requirements. In our country, if ceftazidime were to be used, it is allowed to be used for 24–48 hours, more than that the infectious disease doctor need to be consulted to see if it’s reasonable enough to extend use. Any hospitals could deploy these instructions, depending on how we want to determine our [requirements/measures] …. if there are unjustified use of antibiotics, we could strong feedback. If they have received strong feedbacks many times, there may be some changes*” (Participant 6, senior, medical ward, urban).

“ *I think that there must be a training at provincial hospital level relating to the appropriate use of antibiotics as well as the dangers of the use. It could be twice a year. We should create a chat group to discuss about these topics regularly to make sure that the technical staff/doctors see the importance of the antibiotics. The local hospital can surely do these trainings but it will not be as impactful as having professors from central level to talk about these issues*” (Participant 14, senior, paediatric ward, rural).

“ *All doctors must encourage each other and being a role model. If anybody sees the inappropriate use or overuse of antibiotics, they must give feedback every time*” (Participant 15, junior, intensive care unit, rural).

Many participants also suggested that more advertisement and engagement about the Lao antimicrobial prescribing guidelines as well as enabling easy access with instructions on how to use it (mostly mobile phone application version) could help to improve the guidelines uptake. A few participants said that monitoring and giving feedback on the use could be an option to encourage people to use the guidelines. Improving working hours was brought up among two participants; they argued that long working hours (24 hours on the duty day) was the main obstacle to uptake because people were already tired. Senior prescribers or heads of the wards were frequently mentioned when asking about the key person to support the uptake of the guidelines. Participants suggested that seniors and heads of the ward should support, enforce and monitor their use as well as being role models of guideline usage because junior staff usually followed the decision of the heads. A few participants said that the directors of hospitals should be more proactive and higher levels of civil servants, like the staff of the ministry of health, should get involved as it would be more powerful, and this could gain more trust from the prescribers. Here are some quotations from the study participants:

“ *First, for people to know more about the app, those doctors who use it should advertise it. Second, we need to advertise more, it could be a poster in the elevator if it is allowed. If not, the poster should be put in each ward dashboard therefore people can see it*” (Participant 16, junior, paediatric ward, urban).

“ *We have to use medicine as they [seniors] suggest. If the seniors said that this medicine is better than that medicine, we will have to follow them. If the younger/junior one change [how to prescribe antibiotics appropriately] while the older/senior ones don’t change. We still work for them, therefore we have to follow them*” (Participant 12, junior, intensive care unit, rural).

“ *I really use it [guidelines], my boss encourages me to use it*” (Participant 11, senior, paediatric ward, rural).

“ *Higher level [ministry of health] should recommend the use as wider group of people will know it. It is similar to advertising a product, the more people know about it, the more they use it. Same as this guideline, if there is a recommendation from the high level or from experts in this field, people will trust it*” (Participant 8, senior, intensive care unit, urban).

## Discussion

Findings from this study provide details on how Lao doctors prescribe antibiotics as well as the main challenges to appropriate antibiotic prescribing they face. Participants revealed that they prescribed antibiotics based on several factors. Clinical judgement was the most common factor, followed by availability of existing resources such as laboratory results and antibiotics in their settings. Prescribing based on habit, prescribing due to adopting a precautionary approach ( *e.g.*, concerns about hospital-acquired infection) and following specialist recommendations also contributed to antibiotic prescribing decisions. Data on antimicrobial use in hospitals in Laos were limited until recently ^9, 12^, and information on the prescribing behaviours of Lao doctors was even scarcer. A large antibiotic prescribing behaviour survey of 386 Lao doctors from 25 hospitals across Laos in 2012, showed that half (49%) of participants were confident with their antibiotic prescribing; 22% (86/386) of doctors claimed that their prescribing was driven by antibiotic availability rather than the cause of the infection. In addition, 50% of surveyed participants admitted that patient demands played an important role in their antibiotic prescribing decisions ^18^. A mixed-methods research project aiming to understand how healthcare providers (30 participants) from the Lao capital and a province prescribed antibiotics to pregnant women during their pregnancy and delivery, showed that the majority of participants prescribed antibiotics not only to treat infection or as surgical prophylaxis for caesarean section, but also to prevent post-partum infection in uncomplicated vaginal delivery with episiotomy as a result of a lack in confidence in hygiene in the delivery room ^19^.

Appropriate antibiotic prescribing is a complex topic and involves various factors, especially in low- and middle-income countries such as Laos. This study found that limited access to appropriate antibiotics was the main obstacle to prescribing appropriately. We have described limited access to certain antibiotics in Laos when applying the international antibiotic use guidelines like the WHO AWaRe (access, watch, reserve) antibiotic book in the country. Only 29 of the 39 antibiotics in the AwaRe book were available in Laos with no access to WHO Reserve group antibiotics for Gram negative bacteria resistant to Watch group antibiotics ^20^. Participants in this study mentioned that they have faced unavailability of several Access and Watch group antibiotics in their hospitals on multiple occasions. Long turnaround time of laboratory results was mentioned frequently by study participants. Most participants understood the culture process which could take some days but often they could not switch to recommended antibiotics based on the culture results as a consequence. Microbiological diagnosis in Laos has improved recently thanks to support from the Lao government, and the UK Fleming Fund, with 11 hospitals across the country now able to perform basic conventional cultures with one central hospital and a national centre acting as reference laboratories. Increasing access to microbiological diagnosis is important as well as improving basic knowledge of microbiology among physicians. Other studies have also found that appropriate antibiotic prescribing was influenced by numerous factors such as medical knowledge, experience, awareness, misconceptions, loss of ownership of prescribing decisions, precautionary approaches, expectation of patients and families, workload/lack of staff and available resources ^3, 21, 22^. In our study, most participants claimed that antibiotic demands from patients/families did not affect their prescribing decision. However, some misconceptions of antibiotic effects were found, as some prescribers were reluctant to de-escalate unnecessary broad-spectrum antibiotics already prescribed on another ward before transfer to their ward.

The last edition of the Lao national standard treatment guidelines was released in 2012 which included recommendations for antibiotic therapy for some of the most common infectious diseases. The first antibiotic prescription behaviour survey in Laos conducted in the same year showed that 65% (249/383) of participants preferred local antibiotic prescribing guidelines to the international guidelines but 22% (85/386) mentioned that local guidelines could make their prescribing more challenging rather than supporting it but the authors did not describe the reasons in detail ^18^. Gray *et al*. published the first qualitative study on the uptake of the paediatric guidelines in Laos in 2017. The research team described a number of factors influencing the uptake of the guidelines, for example: comprehension, accessibility (physical availability, language, simplicity, training in use), trust and acceptance, social influences ( *e.g.*, support from seniors, being role models of using guidelines), patient expectations and reinforcement ^13^. This current study explored how Lao prescribers used the first comprehensive antimicrobial prescribing guidelines in the Lao language. The majority of participants said that they were satisfied with the guidelines in terms of the simplicity and accessibility. Most participants admitted that they did not use the guidelines often because they are familiar with most diseases encountered during their practice and they already remembered the guideline recommendation. The participants mentioned that they have also seen their colleagues using the guidelines but not often. Many of them still prescribed based on what they have been doing before. Information about how physicians/prescribers use antibiotic prescribing guidelines or treatment guidelines are not well documented. Beizen *et al.* identified how Australian general practitioners (GPs) accessed and used guidelines to support their antibiotic prescribing decisions. The research team mentioned that GPs’ attitudes played an important role in accessing guidelines. For example, more experienced GPs were less likely to use the guidelines because they claimed that they already knew how to prescribe. The use of the guidelines was also affected by trust and acceptance of the guidelines ( *e.g.*, there might be distrust of the evidence source or concern about economic motives behind the guidelines), patient demands, accessibility ( *e.g.*, cost of the guidelines, ease of access), workload ( *e.g.*, too busy in the practice and no time to update oneself) ^23– 25^. Previous studies have suggested that to be able to improve the uptake of clinical practice/antibiotic use guidelines, the guidelines must be transparent in the development process, comprehensive but simple, easy and free or low cost to access, with training and promotion of their use ^13, 23, 24^. These findings are similar to the themes emerging from this study. In addition, our participants also mentioned that directors of the wards and hospitals should promote and monitor guideline use, and high-level authority figures such as from the ministry of health should get involved to gain more trust from the users.

A limitation of this study was that the main investigator was a key member of the guideline development team. Some participants might therefore not have felt comfortable critiquing the guidelines as much as they wanted to. On the other hand, this could have been seen as a good opportunity for participants to send direct messages to the developer in a constructive way. More female prescribers were interviewed in this study because the majority of the participants from participating wards of these two hospitals were women (69% and 85%). Another limitation was the different medical training background of participants between central and rural hospitals and the fact that not all specialties were represented. Therefore, some information might not be representative of prescribers across the country. It is possible that practice and uptake of guidelines could vary between departments for other reasons such as difference in case-mix; however, we think this was unlikely on these general wards.

Appropriate antibiotic prescription relies on several factors. Findings from this study suggest that improving clinical knowledge of prescribers, assuring easy access to basic and life-saving antibiotics, should be considered. Antibiotic prescribing guidelines could support the rational use of antibiotics and enhance antimicrobial stewardship programmes. To ensure that the guidelines are used regularly, prescribers should be aware of what information they could obtain from the guidelines to support their decision, and recommendations must be updated regularly. Heads of the wards, directors of hospitals and higher authorities should support, monitor and provide feedback on their use to encourage all prescribers to access the guidelines.

## Data Availability

Making the interview transcripts open access risks disclosing the identities of interviewees because the pool of physicians they came from is small. The data are available upon request to the Mahidol-Oxford Tropical Medicine Research Unit Data Access Committee, complying with the data access policy. Queries and applications for data should be directed to datasharing@tropmedres.ac Figshare: Extended data for guideline uptake. https://doi.org/10.6084/m9.figshare.25045091.v1 ^17^. This project contains the following extended data: Extended data_Guidelines uptake_12Jan2024.docx. (Extended data 1: Antimicrobial prescribing behaviours and guidelines uptake discussion guide; and Extended data 2: Demographic characteristics of study participants). Figshare: List of topics in the newly released Lao antimicrobial prescribing guidelines. https://doi.org/10.6084/m9.figshare.26815798.v1 ^26^ This project contains the following extended data: Extended data_List of guidelines topics of paediatric and adult antimicrobial prescribing guidelines.docx (List of topics in the newly released Lao antimicrobial prescribing guidelines). Figshare: Guideline uptake: CORECQ checklist. https://doi.org/10.6084/m9.figshare.26819119.v1 ^27^ This project contains the following extended data: COREQ_checklist.pdf (CORECQ checklist for guideline uptake qualitative study). Data are available under the terms of the Creative Commons Zero "No rights reserved" data waiver (CC0 1.0 Public domain dedication). **Vilada Chansamouth:** Conceptualization, Data Curation, Formal Analysis, Investigation, Methodology, Project Administration, Supervision, Validation, Visualization, Writing – Original Draft Preparation, Writing – Review & Editing **Anousone Douangnouvong:** Data Curation, Investigation, Project Administration, Writing – Review & Editing **Peeyanout Thammavongsa:** Data Curation, Investigation, Writing – Review & Editing **Xaysana Sombandith:** Supervision, Writing – Review & Editing **Sommay Keomany:** Supervision, Writing – Review & Editing **Sommana Rattana:** Supervision, Writing – Review & Editing **Paul N Newton:** Conceptualization, Writing – Review & Editing **Nicholas PJ Day:** Conceptualization, Funding Acquisition, Writing – Review & Editing **Paul Turner:** Conceptualization, Writing – Review & Editing **Mayfong Mayxay:** Conceptualization, Writing – Review & Editing **H. Rogier van Doorn:** Conceptualization, Writing – Review & Editing **Elizabeth A Ashley:** Conceptualization, Methodology, Supervision, Writing – Original Draft Preparation, Writing – Review & Editing
