# Supplementary material for: Understanding hospital antimicrobial prescribing decisions and determinants of uptake of new local antimicrobial prescribing guidelines in Laos
Source: Wellcome Open Res. 2024 Sep 12;9:183. Originally published 2024 Apr 11. [Version 2] doi: 10.12688/wellcomeopenres.20884.2 (PMC11411237; doi:10.12688/wellcomeopenres.20884.2)
Supplement: Peer review history

# Reviewer report: Reviewer response for version 2

**DOI:** 10.21956/wellcomeopenres.25349.r105852

**Published:** 2024-12-06

**Authors:** Minyahil Tadesse Boltena

**Affiliations:** 1 Armauer Hansen Research Institute, Ministry of Health, Addis Ababa, Ethiopia

**Peer Review Report**

**Summary of the Article:**

 The manuscript, *"Understanding hospital antimicrobial prescribing decisions and determinants of uptake of new local antimicrobial prescribing guidelines in Laos [version 2; peer review: 3 approved],"* provides a comprehensive exploration of antimicrobial prescribing behaviors within hospital settings in Laos. The study investigates key factors influencing the adoption of new local antimicrobial prescribing guidelines. Using a qualitative methodology, the authors highlight the decision-making processes, contextual influences, and barriers impacting guideline uptake. This research is particularly relevant given the global push to combat antimicrobial resistance (AMR) through improved antimicrobial stewardship.

 The manuscript is well-structured, cites relevant and up-to-date literature, and offers valuable insights into a critical area of healthcare improvement in low-resource settings. The findings contribute to understanding AMR mitigation efforts and provide actionable recommendations for enhancing guideline adherence in similar contexts.

**Evaluation of the Manuscript:**

1. **Is the work clearly and accurately presented, and does it cite the current literature?**
  - **Yes.**
    The manuscript is clearly written, logically structured, and adheres to scientific standards. It provides a thorough review of current literature on antimicrobial prescribing and guideline implementation, contextualizing the study within the broader field of AMR research. The authors effectively cite studies relevant to both the local context in Laos and the global perspective on antimicrobial stewardship.
2. **Is the study design appropriate and is the work technically sound?**
  - **Yes.**
    The study employs a qualitative design, which is well-suited for exploring prescribing behaviors and guideline uptake. The use of interviews and thematic analysis aligns with the objectives of the study, ensuring that the methodology is robust and technically sound. The authors demonstrate an understanding of qualitative research principles, enhancing the credibility of the findings.
3. **Are sufficient details of methods and analysis provided to allow replication by others?**
  - **Yes.**
    The manuscript provides sufficient detail on participant selection, data collection processes, and analytical techniques. The thematic analysis is clearly described, and the inclusion of representative quotes from participants strengthens the transparency of the findings. These details enable other researchers to replicate the study in similar settings or build upon its findings.
4. **If applicable, is the statistical analysis and its interpretation appropriate?**
  - **Not applicable.**
    As this is a qualitative study, no statistical analysis was performed. The absence of statistical methods is appropriate given the research design.
5. **Are all the source data underlying the results available to ensure full reproducibility?**
  - **Yes.**
    The manuscript includes sufficient supporting data, such as participant quotes and detailed descriptions of the thematic analysis process. These elements allow readers to understand how the conclusions were derived, ensuring reproducibility.
6. **Are the conclusions drawn adequately supported by the results?**
  - **Yes.**
    The conclusions are well-supported by the data and provide a logical interpretation of the findings. The authors effectively tie the results to the study's objectives, highlighting actionable recommendations for improving guideline adoption. The implications for policy and practice are clearly articulated. **Strengths of the Manuscript**
  - Comprehensive exploration of an understudied topic in a low-resource setting.
  - Well-documented methodology and clear presentation of results.
  - Practical recommendations for improving antimicrobial stewardship.

**Suggestions for Improvement:**

 While the manuscript is strong, the following minor improvements could enhance its clarity and impact:

**Discussion of Generalizability**:

 The authors could expand on how the findings may apply to other low-resource settings beyond Laos. A brief comparative discussion would increase the manuscript's relevance to a broader audience.

**Implications for Future Research**:

 Including a dedicated section on potential areas for future research, such as the role of digital tools in supporting guideline adherence, would provide additional value.

**Final Recommendation:**

 This manuscript is well-written, methodologically robust, and provides important insights into antimicrobial prescribing practices. I recommend **acceptance** with minor revisions to enhance generalizability and suggestions for future research. The article makes a valuable contribution to the field of antimicrobial stewardship and is a useful reference for addressing AMR challenges in low-resource settings.

Is the work clearly and accurately presented and does it cite the current literature?

Yes

If applicable, is the statistical analysis and its interpretation appropriate?

Yes

Are all the source data underlying the results available to ensure full reproducibility?

Yes

Is the study design appropriate and is the work technically sound?

Yes

Are the conclusions drawn adequately supported by the results?

Yes

Are sufficient details of methods and analysis provided to allow replication by others?

Yes

Reviewer Expertise:

My name is Minyahil Tadesse Boltena (PhD) is an expert in evidence-based healthcare, specializing in rational antibiotic prescription and the integration of digital health solutions to enhance adherence to antimicrobial treatment guidelines. With a focus on promoting evidence-based clinical practice, I have led initiatives to advance the use of technology for real-time decision support in prescribing practices, particularly in low-resource settings.A strong advocate for aligning clinical and public health policies with high-quality evidence, I have contributed to the production and translation of research evidence to inform policy-making and improve healthcare outcomes. My work emphasizes improving adherence to evidence-based clinical practice guidelines and addressing challenges related to antimicrobial resistance through innovative approaches. With extensive experience in research and implementation, I am dedicated to bridging the gap between clinical research and practical application, ensuring that healthcare policies are both effective and contextually appropriate.

I confirm that I have read this submission and believe that I have an appropriate level of expertise to confirm that it is of an acceptable scientific standard.

# Reviewer report: Reviewer response for version 2

**DOI:** 10.21956/wellcomeopenres.25349.r102398

**Published:** 2024-10-14

**Authors:** Trisha Peel

**Affiliations:** 1 Monash University, Melbourne, Australia

The authors describe a qualitative study investigating end-user uptake and acceptance of the Lao National Antibiotic Prescribing Guidelines. 

 The paper is well structured and presents an in-depth interview with local prescribers from 2 hospitals. 

 There are a few minor points for clarification:

- Please review the following statement for correctness - "Maina *et al*. described that the prevalence of the appropriate use of antibiotics in settings with treatment guidelines **was higher than where guidelines were absent** (14% *vs.* 33%)" Do the authors mean that the prevalence of ***inappropriate use of antibiotics*** was higher...?
- Similarly, for the following statement "The baseline data for **antimicrobial prescription rate** in six hospitals before introduction of the guidelines was 73% .... " The current statement implies that the antimicrobial use increased (albeit by a small percent) after the introduction of the guidelines. If it is purely antibiotic use, is there a measure of whether this was in accordance / compliance with the guidelines?
- How many prescribers are there at the two hospitals? Are the participants representative of the overall prescribing population?
- What are the next steps proposed by the researchers, based on the findings of their study?

Is the work clearly and accurately presented and does it cite the current literature?

Yes

If applicable, is the statistical analysis and its interpretation appropriate?

Not applicable

Are all the source data underlying the results available to ensure full reproducibility?

Yes

Is the study design appropriate and is the work technically sound?

Yes

Are the conclusions drawn adequately supported by the results?

Partly

Are sufficient details of methods and analysis provided to allow replication by others?

Yes

Reviewer Expertise:

Infectious Diseases / Antibiotic Stewardship

I confirm that I have read this submission and believe that I have an appropriate level of expertise to confirm that it is of an acceptable scientific standard.

# Reviewer report: Reviewer response for version 2

**DOI:** 10.21956/wellcomeopenres.25349.r99211

**Published:** 2024-09-18

**Authors:** Soe Yu Naing

**Affiliations:** 1 Utrecht University, Utrecht, The Netherlands

Dear authors,

 Thank you for your thorough responses and for addressing the concerns raised during the review process. The revisions have significantly strengthened the research, making it more insightful and comprehensive.

Is the work clearly and accurately presented and does it cite the current literature?

Partly

If applicable, is the statistical analysis and its interpretation appropriate?

Not applicable

Are all the source data underlying the results available to ensure full reproducibility?

Partly

Is the study design appropriate and is the work technically sound?

Partly

Are the conclusions drawn adequately supported by the results?

Partly

Are sufficient details of methods and analysis provided to allow replication by others?

Partly

Reviewer Expertise:

Antimicrobial resistance, Mixed-method, Microbiology

I confirm that I have read this submission and believe that I have an appropriate level of expertise to confirm that it is of an acceptable scientific standard.

# Reviewer report: Reviewer response for version 1

**DOI:** 10.21956/wellcomeopenres.23108.r89236

**Published:** 2024-08-09

**Authors:** Priyadharsini RP

**Affiliations:** 1 Jawaharlal Institute of Postgraduate Medical Education & Research (JIPMER), Karaikal, India

**Pros:**

 1) A good qualitative study which describes the opinion of the prescribers from various dimensions as how they choose antibiotics.

 2) Challenges was explained with real situation examples.

**Cons:**

 1) Regarding the study design a mixed method study rather than qualitative would have provided more information.

 2)Few references are outdated.

Is the work clearly and accurately presented and does it cite the current literature?

Yes

If applicable, is the statistical analysis and its interpretation appropriate?

Partly

Are all the source data underlying the results available to ensure full reproducibility?

Yes

Is the study design appropriate and is the work technically sound?

Yes

Are the conclusions drawn adequately supported by the results?

Yes

Are sufficient details of methods and analysis provided to allow replication by others?

Yes

Reviewer Expertise:

Antimicrobial resistance, Pharmacovigilance, drug utilization studies.

I confirm that I have read this submission and believe that I have an appropriate level of expertise to confirm that it is of an acceptable scientific standard.

# Response

**Published:** 2024-08-23

**Authors:** Vilada Chansamouth

**Affiliations:** Lao-Oxford-Mahahosot Hospital Wellcome Trust Research Unit, Lao People's Democratic Republic

Thank you for your comments and suggestions. I really appreciate your feedback.

# Reviewer report: Reviewer response for version 1

**DOI:** 10.21956/wellcomeopenres.23108.r81516

**Published:** 2024-05-22

**Authors:** Soe Yu Naing

**Affiliations:** 1 Utrecht University, Utrecht, The Netherlands

The authors examined how clinicians are using the new antimicrobial prescribing guidelines in two general hospitals. They identified antimicrobial prescription behaviors and the perceptions and practices of the clinicians regarding the new guidelines. This is important research, but the manuscript suffers from describing qualitative findings in a quantitative way (e.g., "two participants said that" or "many participants (eight participants) said that"). The authors should seek support from a qualitative researcher to help with writing the results. This manuscript could benefit from addressing the specific comments outlined below.

 Scientific Comments

1. The authors did not follow any guidelines for reporting qualitative findings. I suggest that the authors follow the COREQ (Consolidated Criteria for Reporting Qualitative Research) Checklist and include the checklist in the resubmission.
2. In the introduction, the baseline data for antimicrobial prescription rates were 73% (inpatient) and 27% (outpatient) in 2021, and 76.9% (inpatient) and 30% (outpatient) in 2022. However, the dashboard showed different rates (74.3% (inpatient), 24.7% (outpatient) in 2021 vs. 72.9% (inpatient) and 26.4% (outpatient) in 2022). Which one is correct? Justify this discrepancy.
3. If your statement is correct (refer to comment#2), explain why the introduction of the guidelines increased antimicrobial prescription.
4. Given that this is a qualitative study, the discussion of sample size should be based on an iterative process rather than numerical guidance for determining an effective sample size for qualitative research. The iterative process of sampling was not clearly explained. Explain how you arrived at 16 participants.
5. For each quotation, include the participant's role, affiliated ward, and whether they are from an urban or rural hospital.
6. Remove the sentence in the Results section stating, "Theoretical saturation was reached after 16 interviews." This cannot be stated without triangulation and achieving maximum variation.
7. Given that the clinicians were selected from five different wards, it is important to discuss differences in practices and uptake among these wards. Include this in the results and discussion sections.
8. In Table 2, some participants reported using the app, whereas others used the book. It was not clearly described who had access to the mobile app or the pocketbook. Given that this RCT (LAMPA) compared the proportion of antimicrobial prescriptions based on a mobile phone application versus a pocketbook, it is important to know whether the mobile app or pocketbook increased the uptake of guidelines. Include this in the results and discussion sections.

General Comments

1. Please include the Lao comprehensive antimicrobial prescribing guidelines (if possible, in English) in the extended data.
2. There is a typo in the discussion (second paragraph, line 13): "Whatch" should be "Watch."
3. Proofread for grammar, punctuation, and typos.

Is the work clearly and accurately presented and does it cite the current literature?

Partly

If applicable, is the statistical analysis and its interpretation appropriate?

Not applicable

Are all the source data underlying the results available to ensure full reproducibility?

Partly

Is the study design appropriate and is the work technically sound?

Partly

Are the conclusions drawn adequately supported by the results?

Partly

Are sufficient details of methods and analysis provided to allow replication by others?

Partly

Reviewer Expertise:

Antimicrobial resistance, Mixed-method, Microbiology

I confirm that I have read this submission and believe that I have an appropriate level of expertise to confirm that it is of an acceptable scientific standard, however I have significant reservations, as outlined above.

# Response

**Published:** 2024-08-23

**Authors:** Vilada Chansamouth

**Affiliations:** Lao-Oxford-Mahahosot Hospital Wellcome Trust Research Unit, Lao People's Democratic Republic

Thank you for your comments. We really appreciate your expert feedback and suggestions. We have revised the content to present the information more qualitatively, retaining participant numbers only in the tables.

 1. We have followed the CORECQ checklist, which i have shared it as the extended data.

 2. The percentages are correct. we have added clarification in the text to explain where these numbers came from. To obtain the 73% for inpatient prescriptions and 27% for outpatient prescriptions, the first survey round of 2021 (2021-Q1) should be selected, as this survey was conducted before the guideline implementation. One year later, in the first survey round of 2022 (2022-Q1), the figures were 76.9% for inpatient prescriptions and 30% for outpatient prescriptions. Please see the amendment in the main text “The baseline data for antimicrobial prescription rate in six hospitals before introduction of the guidelines was 73% (95% CI, 70%-76.5%) among inpatients and 27% (95% CI, 25.8%-28.9%) among outpatients in the first survey round of 2021, the antimicrobial prescribing percentage increased to 79.6% (95% CI, 75.7%-83%; p=0.02), and 30% (95% CI, 28.4%-32.2%; p=0.01) among in- and outpatients, respectively, one year (the first survey round of 2022) after the introduction of the guidelines”.

 3. One objective of this study is to understand why antimicrobial prescriptions did not improve after the guideline implementation. We have added potential reasons for this increase in the introduction, which are further explored in the findings. Please see at the end of the introduction section: “One probable reason could be due to the Covid-19 pandemic in Laos during 2021 and 2022. An increase of antibiotic use was described globally during Covid-19 pandemic ^26^. Therefore, this study aimed to explore the determinants of antibiotic prescribing decisions and how these new Lao antimicrobial prescribing guidelines were used.”

 4. We have amended this section. Please see “Participants” section on the methods: “Participants in this study were purposively selected based on seniority and their specialties (prescribing antibiotics, from infectious disease departments or intensive care units, pediatric *vs.* adult). We focused on prescribers who prescribed antibiotics at least three times a week on average and who had access to the local antimicrobial prescribing guidelines, either pocketbook or mobile phone application versions or both, and were willing to give written informed consent to participate. The possible target prescribers were listed based on the above-mentioned criteria before the invitations were distributed. The purposive selection allowed us to capture a range of different perspectives. The eligible participants were invited to join the in-depth interviews, including senior doctors and juniors/residents in a 1:1 ratio. There is no formal definition of junior and senior physicians in Laos. In general, doctors who are less than 40 years old, are classified as early career, 40–50 years old as mid-career and >50 years old as late career. It is more complex when it comes to “senior *vs.* junior”. In this study, junior was defined as doctors/prescribers aged <40 years old or with medical experience of 10 years or less. Senior was defined as doctors/prescribers aged ≥40 years old and with medical experience of more than 10 years.”

 5. We have amended as suggested

 6. Thank you for suggestions, it has been removed

 7. Thank you for your comments. Overall, we found that most doctors from this study had similar opinions in how they prescribed antibiotics and when they accessed the newly released guidelines. We did not get any impression of different practices or uptake. Our point prevalence survey (PPS) data suggests there are no major differences in case mix although it is possible more complicated patients are seen at central level. We focused mostly on central and provincial level rather than ward level due to small number of interviewees from several wards. We have added this to the discussion section “It is possible that practice and uptake of guidelines could vary between departments for other reasons such as difference in case-mix; however we think this was unlikely on these general wards.”

 8. Thank you for suggestion. We focused on participants who had access to either paper-based or app-based guidelines or both as mentioned in the methods. At the end, we found that nearly all of them had access to both (15 participants out of 16). We have added this information in the results. Please see at the end of the first paragraph of the results “Nearly all participants had access to both paper-based and app-based Lao antimicrobial prescribing guidelines, with only one participant having access solely to the app-based guideline” The LAMPA study had not completed yet when conducting this in-depth interview. The results of LAMPA will be published in a different manuscript.

 General Comments:

 1. There is no English version. We have added the list of guideline topics for both paediatric and adult versions in the extended data

 2. Amended

 3. Done
